# Supplementary material for: Periodontal Indices as Predictors of Cognitive Decline: Insights from the PerioMind Colombia Cohort
Source: Biomedicines. 2025 Jan 15;13(1):205. doi: 10.3390/biomedicines13010205 (PMC11760870; doi:10.3390/biomedicines13010205)
Supplement: Supplementary file 1 [file biomedicines-13-00205-s001.zip › biomedicines-3411802-supplementary/Supplementary_tables.pdf]

Specific Periodontal Index Alterations in Elders with Mild Cognitive Impairment: Insights from the PerioMind Colombia Cohort

Catalina Arévalo-Caro, Diego López, Jose Antonio Sánchez Milán, Cristina Lorca, María Mulet, Humberto Arboleda, Sergio Losada Amaya, Aida Serra †

and Xavier Gallart-Palau †

† Xavier Gallart-Palau and Aida Serra are joint senior authors.

Summary table:

Content	*Page*
Supplementary Table S1. *Naïve Bayes model predictive results for Control and mild cognitive impairment (MCI) groups in the PerioMind Colombia cohort.*	*S-2*
Supplementary Table S2. *Partial Least Squares Discrimination (PLSD) model predictive results for Control and mild cognitive impairment (MCI) groups in the PerioMind Colombia cohort.*	*S-3*
Supplementary Table S3. *Predictive model performance of the Naïve Bayes and Partial Least Squares Discriminant (PLSD) models in predicting cognitive status (Control or mild cognitive impairment (MCI)) among participants in the PerioMind Colombia cohort.*	*S-4*

Supplementary table S1. Naïve Bayes model predictive results for Control and mild cognitive impairment (MCI) groups in the PerioMind Colombia cohort. Parameters analyzed include demographic (age, sex), educational level, and periodontal variables such as stages of periodontal disease (EFP/AAP and CDC/AAP classifications), periodontal disease presence (CDC/AAP), gingival redness, and pocket depth (PD ≥ 4 mm). Values are presented as mean ± standard deviation (SD).

	**Control**	**MCI**
**Age**	70 ± 5,25	69,8 ± 3,19
**Sex**	0,35 ± 0,49	0,3 ± 0,48
**Basic Educational level**	1,95 ± 0,82	1,7 ± 0,67
**Stages Periodontal disease (EFP/AAP)**	3,4 ± 0,75	3,6 ± 0,52
**Stages Periodontal disease (CDC/AAP)**	2,25 ± 0,91	2,5 ± 0,53
**Periodontal disease (CDC/AAP)**	0,9 ± 0,31	1 ± 0
**Gingival redness**	0,14 ± 0,26	0,43 ± 0,15
**Pocket Depth (PD) ≥ 4 mm**	0,10 ± 0,2	0,27 ± 0,27

Supplementary table S2. Partial Least Squares Discrimination (PLSD) model predictive results for Control and mild cognitive impairment (MCI) groups in the PerioMind Colombia cohort. Parameters analyzed include demographic (age, sex), educational level, and periodontal variables such as stages of periodontal disease (EFP/AAP and CDC/AAP classifications), periodontal disease presence (CDC/AAP), gingival redness, and pocket depth (PD ≥ 4 mm). Values are presented as mean ± standard deviation (SD).

	**Control**	**MCI**
**Age**	0.1870742	-0.18707424
**Sex**	-0.12761256	0.12761258
**Basic Educational level**	-	-
**Stages Periodontal disease (EFP/AAP)**	-	-
**Stages Periodontal disease (CDC/AAP)**	-0.061256085	0.06125609
**Periodontal disease (CDC/AAP)**	-0.14068814	0.14068815
**Gingival redness**	-0.18733318	0.18733321
**Pocket Depth (PD) ≥ 4 mm**	-0.04541736	0.04541737

Supplementary table S3. Predictive model performance of the Naïve Bayes and Partial Least Squares Discriminant (PLSD) models in predicting cognitive status (Control or mild cognitive impairment (MCI)) among participants in the PerioMind Colombia cohort. The table shows the number of correctly and incorrectly classified individuals for each group (True Control and True MCI), along with the accuracy percentages for each model. The Naïve Bayes model achieved 100% overall accuracy, while the PLSD model demonstrated 96.7% accuracy.

	**Naïve Bayes Model**			**Partial Least Squares Discrimination (PLSD) Model**		
	**Predicted Control**	**Predicted MCI**	**Accuracy (%)**	**Predicted Control**	**Predicted MCI**	**Accuracy (%)**
**True Control**	20	0	100	19	1	95
**True MCI**	0	10	100	0	10	100
**Overall Accuracy (%)**	100			96.7		
